# POSTAR2: deciphering the post-transcriptional regulatory logics

**DOI:** 10.1093/nar/gky830

**Published:** 2018-09-17

**Authors:** Yumin Zhu, Gang Xu, Yucheng T Yang, Zhiyu Xu, Xinduo Chen, Binbin Shi, Daoxin Xie, Zhi John Lu, Pengyuan Wang

**Affiliations:** 1 MOE Key Laboratory of Bioinformatics, Center for Synthetic and Systems Biology, School of Life Sciences, Tsinghua University, Beijing 100084, China; 2 Division of General Surgery, Peking University First Hospital, Beijing 100034, China; 3 Department of Molecular Biophysics and Biochemistry, Yale University, New Haven, CT 06520, USA

## Abstract

Post-transcriptional regulation of RNAs is critical to the diverse range of cellular processes. The volume of functional genomic data focusing on post-transcriptional regulation logics continues to grow in recent years. In the current database version, POSTAR2 (http://lulab.life.tsinghua.edu.cn/postar), we included the following new features and data: updated ∼500 CLIP-seq datasets (∼1200 CLIP-seq datasets in total) from six species, including human, mouse, fly, worm, *Arabidopsis* and yeast; added a new module ‘Translatome’, which is derived from Ribo-seq datasets and contains ∼36 million open reading frames (ORFs) in the genomes from the six species; updated and unified post-transcriptional regulation and variation data. Finally, we improved web interfaces for searching and visualizing protein–RNA interactions with multi-layer information. Meanwhile, we also merged our CLIPdb database into POSTAR2. POSTAR2 will help researchers investigate the post-transcriptional regulatory logics coordinated by RNA-binding proteins and translational landscape of cellular RNAs.

## INTRODUCTION

RNA-binding proteins (RBPs) control every aspect of post-transcriptional regulatory logics, including maturation, localization, degradation, modification, editing and translation of cellular RNAs (1–3). Several high-throughput sequencing technologies exist for determining RBP-binding sites and translational dynamics *in vivo*, most notably ultraviolet crosslinking followed by immunoprecipitation and sequencing (CLIP-seq) (4,5) and ribosome profiling (Ribo-seq) (6). In recent years, CLIP-seq and Ribo-seq have been widely used to decipher the post-transcriptional regulatory logics coordinated by RBPs and translational landscape of cellular RNAs in various species.

CLIP-seq studies have identified RBP-binding sites from a broad set of cell and tissue types from various species (7,8). In addition, large amounts of gene expression profiles, RNA modification sites, RNA editing sites, as well as disease-associated variants, have been identified attributed to efforts on large-scale genomics studies and development of bioinformatics algorithm. The regulatory mechanisms of RBP-binding sites underlie diseases and phenotypes can be revealed by combining information from RBP binding, other post-transcriptional regulatory events and genomic variation. Ribo-seq is a powerful technology for measuring translation efficiency by mapping the ribosome-binding positions across the transcriptome at a sub-codon resolution (6,9). Previous studies have shown that translation efficiency and translational dynamics can be regulated by RBP binding (2,10,11). However, the integration of these large-scale datasets for the exploration of the coupling between post-transcriptional and translational regulation remains a great challenge.

Here, we developed POSTAR2 by systematically identifying RBP-binding sites derived from more CLIP-seq datasets, and predicting open reading frames (ORFs) using larger-scale Ribo-seq datasets from six species, including human, mouse, fly, worm, *Arabidopsis* and yeast. POSTAR2 provides an updated interactive user interface for searching and visualizing RNA–protein interactions and ORFs from various tissue types, cell lines, developmental stages and conditions. Moreover, by integrating microRNA (miRNA)-binding sites, RNA modifications sites, RNA editing sites, single nucleotide polymorphisms (SNPs), genome-wide association study (GWAS) variants and cancer somatic mutations, POSTAR2 can be used to explore the potential associations between RBP-binding sites and these data. POSTAR2 made significant improvements in data collection from more species, and could be useful for investigating the post-transcriptional regulatory logics coordinated by RBPs, as well as translational landscape of cellular RNAs.

## DATA COLLECTION AND PROCESSING

### Collection of CLIP-seq datasets

**Figure 1.**
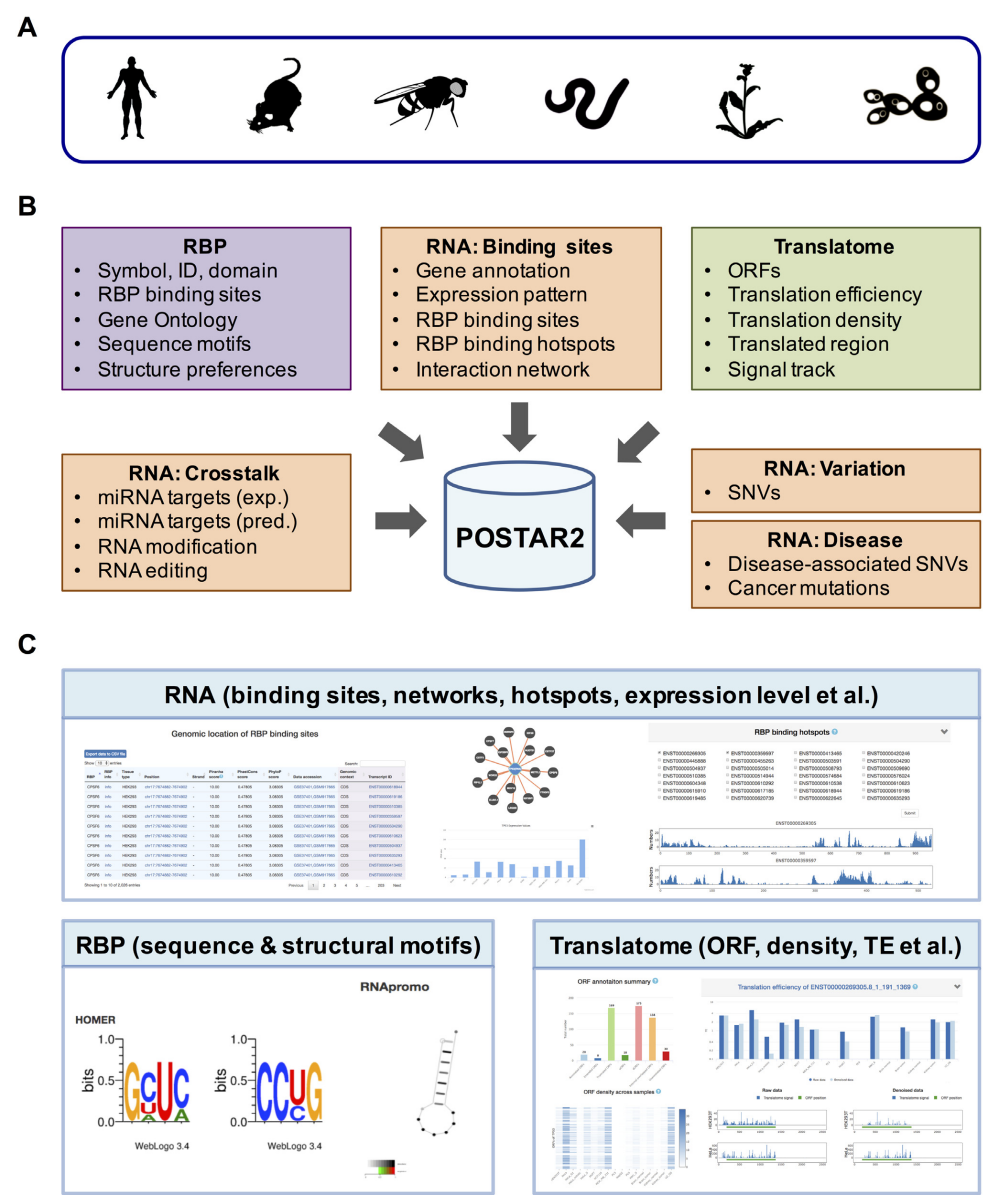
Framework to construct POSTAR2 database. (**A**) POSTAR2 covers six species including human, mouse, fly, worm, *Arabidopsis* and yeast. (**B**) POSTAR2 provides three modules: (i) ‘RBP’ module, which provides annotations and functions of RBPs, as well as RBP-binding sites; (ii) ‘RNA’ module, consisting of several sub-modules including ‘Binding sites’, ‘Crosstalk’, ‘Variation’ and ‘Disease’, which annotates the RBP-binding sites using various regulatory events and genomic variants; (iii) ‘Translatome’ module, which aims for exploring the translation landscape of genes across different tissues and cell lines. (**C**) POSTAR2 provides a user-friendly interface for searching and visualization such as table views, network views, histograms and heatmaps.

**Figure 2.**
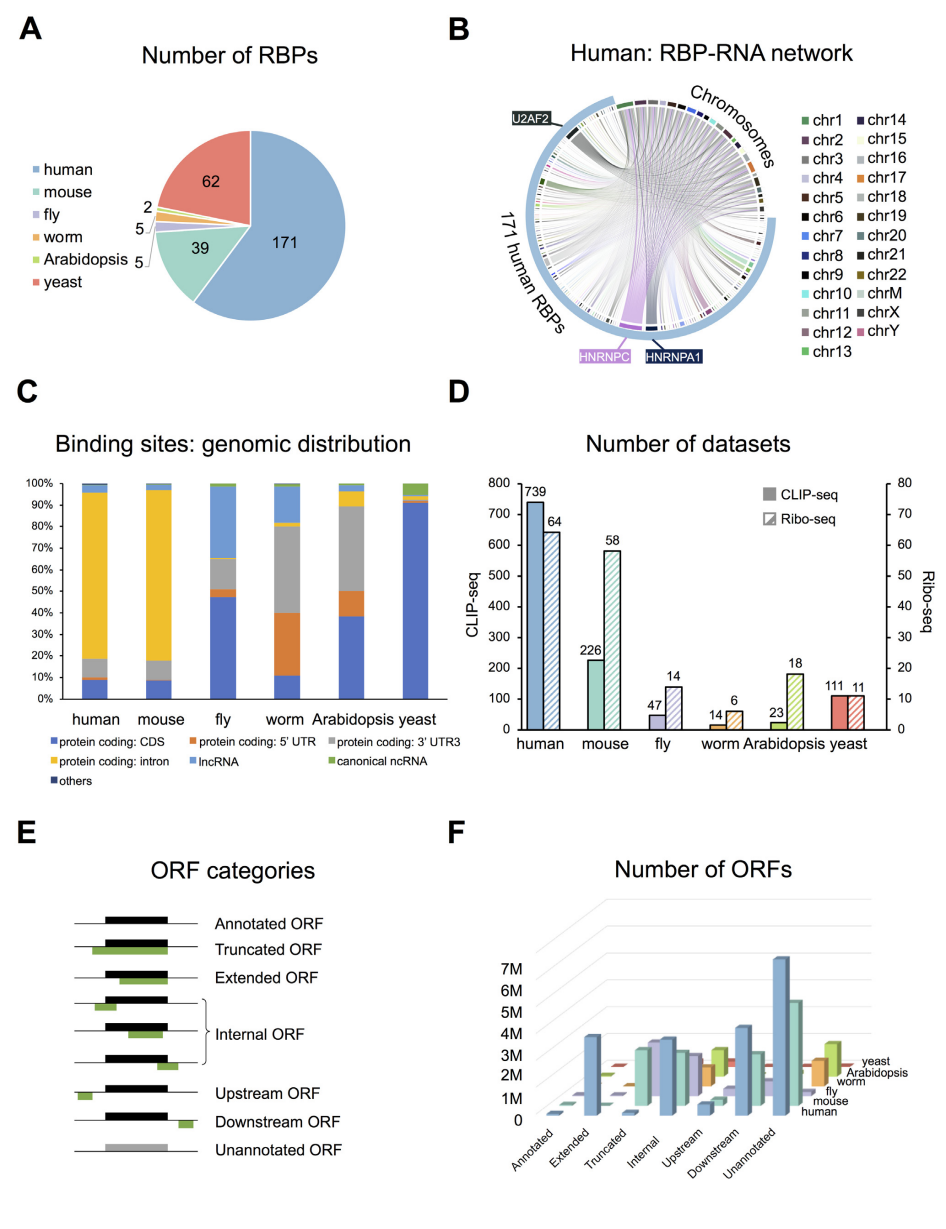
Statistics of POSTAR2 database. (**A**) Number of RBPs in the human, mouse, worm, fly, *Arabidopsis* and yeast. (**B**) The distribution of human RBP-binding sites on chromosomes. HNRNPC, HNRNPA1 and U2AF2 have the largest number of binding sites among 171 human RBPs. (**C**) Genomic distribution of RBP-binding sites in six species identified using Piranha. (**D**) Summary of CLIP-seq and Ribo-seq datasets. (**E**) Diagram for different ORF categories. (i) Annotated ORFs (aORFs): ORFs that are annotated by reference annotation, which are colored with black in the diagram. (ii and iii) Truncated and extended ORFs: ORFs that contain the same stop codon with aORFs, but have different translation initiation sites. (iv) Internal ORFs: ORFs that are located in or have partial overlap with aORFs. (v and vi) Upstream and downstream ORFs: ORFs that are located upstream or downstream of aORFs. (vii) Unannotated ORFs: ORFs that are defined from transcripts without any reference annotation. (**F**) Number of ORFs for each category across six species.

POSTAR was developed to house and distribute RBP-binding sites from human and mouse (12). To expand and update our database, we manually collected newly published CLIP-seq data from the Gene Expression Omnibus (GEO) and Sequence Read Archive (SRA) databases (13). At present, POSTAR2 contains a large set of RBP-binding sites derived from CLIP-seq datasets and covers six species, including human, mouse, worm, fly, *Arabidopsis* and yeast (Figure 1 and Table 1). We first obtained the processed datasets in human and mouse from POSTAR (12), and the processed datasets in worm and yeast from CLIPdb (7). In addition, we collected 298 new datasets of the six species from recent publications. We also updated 332 eCLIP-seq datasets released by the ENCODE consortium (14,15). In total, POSTAR2 contains 1160 CLIP-seq datasets, which cover 284 RBPs from six species (Figure 2A). To our knowledge, this is the largest collection of RBP-binding sites identified from various CLIP-seq technologies, including HITS-CLIP, PAR-CLIP, iCLIP, eCLIP and PIP-seq (Supplementary File S1 and Supplementary File S2).

**Table 1.** Overview of data curated in POSTAR2

	Category	Human	Mouse	Fly	Worm	Arabidopsis	Yeast	Notes
RBP-binding sites	RBP-binding sites from experiments	3 759 076	1 193 757	97 322	35 652	31 183	324 641	All CLIP-seq peaks called by Piranha
		75 734	110 876	1717	46	568	5800	HITS-CLIP peaks called by CIMS
		15 788 784	226 458	417 150	29 784	NA	4 575 287	PAR-CLIP peaks called by PARalyzer
		9 131 076	1 067 309	87 049	406 571	119 754	NA	iCLIP peaks called by CITS
		2 436 040	NA	NA	NA	NA	NA	eCLIP peaks called by ENCODE
		439 817	NA	NA	NA	NA	NA	PIP-seq peaks called by PMID24393486
RBP	RBPs	171	39	5	5	2	62	Ensembl, PMID25365966
	Sequence motifs	1218	252	30	30	12	366	MEME, HOMER
	Structural preferences	1169	245	30	30	11	352	RNApromo, RNAcontext
	Gene Ontologies	108 787	41 501	2976	2145	1238	26 013	GOBP, GOMF, GOCC
RNA	Gene expression	12 cell/tissue types	10 cell/tissue types	30 developmental stages	35 developmental stages	4 cell/tissue types	3 conditions	GEO database Expression Atlas
	miRNA-binding sites from experiments	3 906 955	1 588 861	NA	NA	NA	NA	AGO CLIP-seq peaks called by Piranha, the targeting miRNAs identified by miRanda
	miRNA-binding sites from predictions	12 196 959	7 563 080	1 099 046	671 012	2524	NA	miRanda, RNAhybrid, psRobot, psRNAtarget
	RNA modification sites	489 629	495 232	6819	NA	20 331	71 466	RMBase2, PMID26863196
	RNA editing sites	2 583 302	8846	5037	111 134	NA	NA	RADAR, DARNED, PMID25373143
	SNVs	323 138 224	81 432 271	5 618 672	189 322	13 412 332	486 302	dbSNP, PMID21079745
	GWAS SNPs	278 473	NA	NA	NA	NA	NA	GWASdb2
	Clinically important SNPs	131 919	NA	NA	NA	NA	NA	ClinVar
	Cancer TCGA whole-exome SNVs	3 427 854	NA	NA	NA	NA	NA	PMID29596782
	Cancer TCGA whole-genome SNVs	4 745 891	NA	NA	NA	NA	NA	PMID23945592
	Cancer COSMIC SNVs	2 371 219	NA	NA	NA	NA	NA	COSMIC
Translatome	Condition	17 cell/tissues types	6 cell/tissue types	5 stages/cell types	3 cell types	8 conditions	6 conditions	GEO database
	Annotated ORF	65 319	38 686	30 357	20 108	26 916	6498	ORFs annotated by reference
	Truncated ORF	2 922 855	2 072 685	1 993 300	556 378	749 484	193 126	ORFs with the same stop codon as aORF but downstream start codon
	Extended ORF	102 866	3128	29 440	7840	11 673	0	ORF with the same stop codon as aORF but upstream start codon
	Internal overlapped ORF	2 828 307	1 973 410	1 490 433	704 924	983 723	193 982	Off-frame ORFs that overlaps with aORF
	uORF	413 508	226 589	273 310	14 460	48 784	0	ORFs located upstream of aORF
	dORF	3 266 469	1 921 443	551 813	51 642	141 319	0	ORFs located downstream of aORF
	Unannotated ORF	5 815 149	3 836 094	155 945	953 269	1 210 658	11 461	ORFs with no annotation

### Identification of RBP-binding sites

For the newly collected CLIP-seq datasets, we used the uniform preprocessing pipeline from CLIPdb (7) to preprocess the raw data. Briefly, we first trimmed the adaptor sequences from the raw reads using FASTX-Toolkit package (http://hannonlab.cshl.edu/fastx_toolkit). We only retained reads with quality score above 20 in 80% of their nucleotides. The reads shorter than 13 nt after adaptor trimming were discarded. Finally, we collapsed identical reads to minimize polymerase chain reaction duplicates.

After preprocessing, the retained reads were aligned to their respective genomes using Bowtie (16) and NovoAlign (http://www.novocraft.com). Notably, to make the genomic coordinates of the binding sites consistent between the newly collected data and available data in POSTAR, we used the same genome versions in POSTAR for read alignment, i.e. human (hg19) and mouse (mm10), together with the genomes for four additional species, i.e. worm (ws235), yeast (R64-1-1), fly (dmel-r6.18) and *Arabidopsis* (TAIR10). We then used both CLIP technology-specific and non-specific tools to identify binding sites for each dataset, respectively. Briefly, we used Piranha (17) to identify binding sites for HITS-CLIP, PAR-CLIP and iCLIP datasets with parameter -b 20 -d ZeroTruncatedNegativeBinomial -p 0.01. We also applied CLIP technology-specific tools for binding site identification with default parameters: using PARalyzer (18) for PAR-CLIP datasets, using CIMS (19) for HITS-CLIP datasets and using CITS (a module in CIMS software) (19,20) for iCLIP datasets. The binding site coordinates from HITS-CLIP, PAR-CLIP, iCLIP and PIP-seq, which are human genome hg19-based, were converted to hg38 using the UCSC liftOver tool. As for eCLIP, the hg38-based binding sites were directly downloaded from the ENCODE data portal (https://www.encodeproject.org/, NOV 2017). Finally, we identified millions of RBP-binding sites, and visualized the RBP–RNA interaction network in human (Figure 2B).

### Annotation of RBPs and RBP-binding sites

For each RBP, we obtained the information of RNA-binding domains from Pfam database (21). We also collected GO term annotations of RBPs from AmiGO (22). We annotated RBP-binding sites using their respective genome annotations (human, Gencode V27; mouse, Gencode VM7; fly, Flybase dmel-r6.18; worm, WormBase ws235; *Arabidopsis*, TAIR10; yeast, SGD R64-1-1) (23–27). To enable systematic annotation of RBP-binding sites in long non-coding RNAs (lncRNAs), we used lncRNA annotations from Gencode (23) for human and mouse, and lncRNA annotations from NONCODE 2016 (28) for fly, worm, *Arabidopsis* and yeast. The distribution of genomic elements for RBP-binding sites showed difference between species (Figure 2C). We found that human and mouse exhibited similar patterns of genomic elements, suggesting the conservation of functional RBP binding between mammals.

We collected RNA-seq datasets from the 12 human cell/tissue types and 10 mouse cell/tissue types that are used in the CLIP experiments (Supplementary File S3), and mapped the reads using TopHat (29), followed by estimating the expression level of the genes using Cufflinks (30). For the 30 developmental stages from fly, 35 developmental stages from worm, 4 tissue types from *Arabidopsis* and 3 conditions (wild-type, glucose starvation and nitrogen starvation) for yeast, we obtained the gene expression data from the Expression Atlas (31) and our previous paper (32). We prepared and intersected miRNA-binding sites, RNA modification sites, RNA editing sites, SNPs and disease-associated variants with RBP-binding sites according to the same computational pipeline used in POSTAR (12). The coordinates of these genomic regions for human build hg19 were also converted to hg38 using the UCSC liftOver tool.

We used the same strategy from POSTAR (12) to predict sequence motifs and structural preferences of RBP-binding sites. Briefly, the binding sites from each CLIP-seq sample were separated into independent training and testing set. Then, we used MEME (33) and HOMER (34) to identify and report up to five sequence motifs in the training set. Next, we calculated the enrichment for the initially detected motifs in the testing set using FIMO (35) and selected the three most enriched sequence motifs. The sequence motifs were visualized using WebLogo (36). To predict structural preferences of RBP-binding sites, the binding sites from each CLIP-seq sample were extended to at least 60 nt in length. We then used RNAcontext (37) to detect local structural motifs. The structural annotation used in RNAcontext included paired (P), hairpin loop (L), bulge/internal/multi-loop (M) and unstructured (U). In addition, we used RNApromo (38) to predict structural elements that are enriched within the RBP-binding sites (*P*-value <0.05).

### Ribo-seq datasets collection and ORF identification

We collected 171 Ribo-seq datasets as well as matched RNA-seq datasets from the six species from the GEO and SRA databases (13) for translation efficiency (TE) calculation (Figure 2D; Supplementary File S4 and Supplementary File S5). For each Ribo-seq dataset, we overlapped with the annotated start codon and calculated its 5′ distance to the first nucleotide of annotated start codons to infer the positions of peptidyl-site (P-site) for each read length. Thereafter, we applied this offset to represent the P-sites positions of all the reads that are of the same length and generated a P-site signal track for all transcripts based on the inferred P-sites positions for mapped reads.

For each species, the ORFs were predicted by scanning the transcript sequence in which we defined any possible AUG start codon pairing with nearest in-frame stop codon (UAA, UAG and UGA) as an ORF. ORFs shorter than 300 nt were defined as small ORFs (sORF). All predicted ORFs are further categorized into different subtypes according to their relative position with the aORFs (Figure 2E). In total, we identified ∼36 million ORFs among the six species, and numbers of ORFs showed the difference between different categories among six species (Figure 2F). To identify translated ORFs across different tissue types, cell lines, developmental stages and conditions, we used several computational tools, including RiboWave (39), RiboTaper (40), ORFscore (41) and RibORF (42), to detect pattern of 3-nt periodicity within each ORF, as well as the uneven distribution among different reading frames while translating. Default parameters were used for these tools.

### Translation efficiency and translation density calculation

Translation efficiency (TE) measures the rate of messenger RNA translated into proteins, which can be estimated as the ratio between RPKM values of Ribo-seq and RNA-seq (6). We calculated TE under different tissue types, cell lines, developmental stages and conditions. We used either original signal of Ribo-seq (raw data) or denoised periodic footprint by RiboWave (39) (denoised data) as the estimation of ribo-seq signal strength.

Translation density is determined by normalizing the abundance of Ribo-seq reads along the studied ORF with the length of ORF to estimate the intensity of the ORF. We calculated translation density using both raw data (original ribo-seq signal) and denoised data (RiboWave-derived footprint) as input, and presented the results in both methods.

### Database architecture

All data in POSTAR2 were processed and stored into a MySQL Database (version 5.6.39). The client-side user interface was implemented by the HTML5 and JavaScript libraries, including jQuery (http://jquery.com) and Bootstrap (http://getbootstrap.com). The server-side was used PHP scripts (version 5.6.39) and JavaScript. Plots of query results in POSTAR2 were generated by plotly.js library (https://plot.ly) and Highcharts (https://www.highcharts.com). Tables of query results were produced by the DataTables JavaScript library (https://www.datatables.net) that allows users to search and sort results. Visualization was implemented using the UCSC Genome Browser. We have tested web in several popular browsers including Google Chrome, Safari, Internet Explorer and Firefox.

## DATABASE FEATURES AND APPLICATIONS

### Web interface

POSTAR2 provides a user-friendly interface for searching and visualizing protein–RNA interactions with multi-layer information of post-transcriptional regulation, disease-associated variation, as well as translation landscape of RNAs. POSTAR2 contains three modules (Figure 1B): (i) ‘RBP’ module; (ii) ‘RNA’ module, consisting of several sub-modules including ‘Binding sites’, ‘Crosstalk’, ‘Variation’ and ‘Disease’ and (iii) ‘Translatome’ module. Here, we briefly introduce each module below.

The ‘RBP’ module provides various annotations for the RBPs, including RNA recognition domains, RBP ontology, sequence motifs and structural preferences, as well as all the binding sites for the query RBP and enriched GO terms for the target genes (Figure 1C, lower-left panel).

As for the ‘RNA’ module (Figure 1C, upper panel), the ‘Binding sites’ sub-module provides all of the RBP-binding sites of the target gene, regardless of different CLIP-seq technologies or different peak calling methods. Furthermore, table and network view present the interaction of RBPs and target genes. We also collected multiple annotations for the target gene including genomic location, associated diseases, as well as expression patterns across different cell lines, tissue types, developmental stages or conditions. In addition, we defined ‘RBP-binding hotspots’ to decode number of binding proteins of each 20-nt bin along RNA’s precursor, which delivers an overview of the RBP binding hot regions of each RNA’s precursor to users. The ‘Crosstalk’ sub-module provides the interactions of RBP-binding sites and post-transcriptional regulations including miRNA targets, RNA modification and RNA editing (Figure 1B). RBPs participate in various steps and play vital roles in most post-transcriptional regulation processes so that users can investigate potential crosstalk of these regulatory events in this module. To understand how various genomic variants affect RBP binding and cooperate to orchestrate post-transcriptional regulation, the ‘Variation’ sub-module and the ‘Disease’ sub-module integrate SNVs and disease-associated SNVs to provide insights into the causal SNVs underlying regulatory mechanisms and human diseases (Figure 1B).

In addition to the above two modules, we also built a new module ‘Translatome’ for characterizing the translation landscape of RNAs (Figure 1C, lower-right panel). Users can choose a species (e.g. human, mouse, fly, worm, *Arabidopsis* or yeast) and input a gene name to search within. POSTAR2 returns a summary frame and three tables, the summary frame contains a histogram shows the number of ORFs in different categories and a heat map provides the density of each ORF across various samples. These three tables present aORFs, extended/truncated ORFs and other ORFs, respectively, and each ORF is labeled according to the transcript ID, the relative reading frame of the ORF, the translation start site and termination site. Users can also sort ORFs by length in these tables to screen out sORF that are shorter than 300 nt. Moreover, each ORF ID provides a link for more details about the translation pattern of this ORF, including translation efficiency, translation density and identified translated region of the ORF. The column diagram provides visualization to compare translation state of the ORF across different tissue types, cell lines, developmental stages or conditions. In addition, users can select their interested conditions to simultaneously visualize signal tracks of each ORF along its located transcript.

### Example applications

We designed a user-friendly interface, which provides a platform to connect protein–RNA interactions with multi-layer information of post-transcriptional regulation and disease-associated variants, as well as translation landscape of RNAs. Here, we illustrate an example application with ADAM17 to demonstrate how to explore potential regulatory mechanism underlies human diseases.

**Figure 3.**
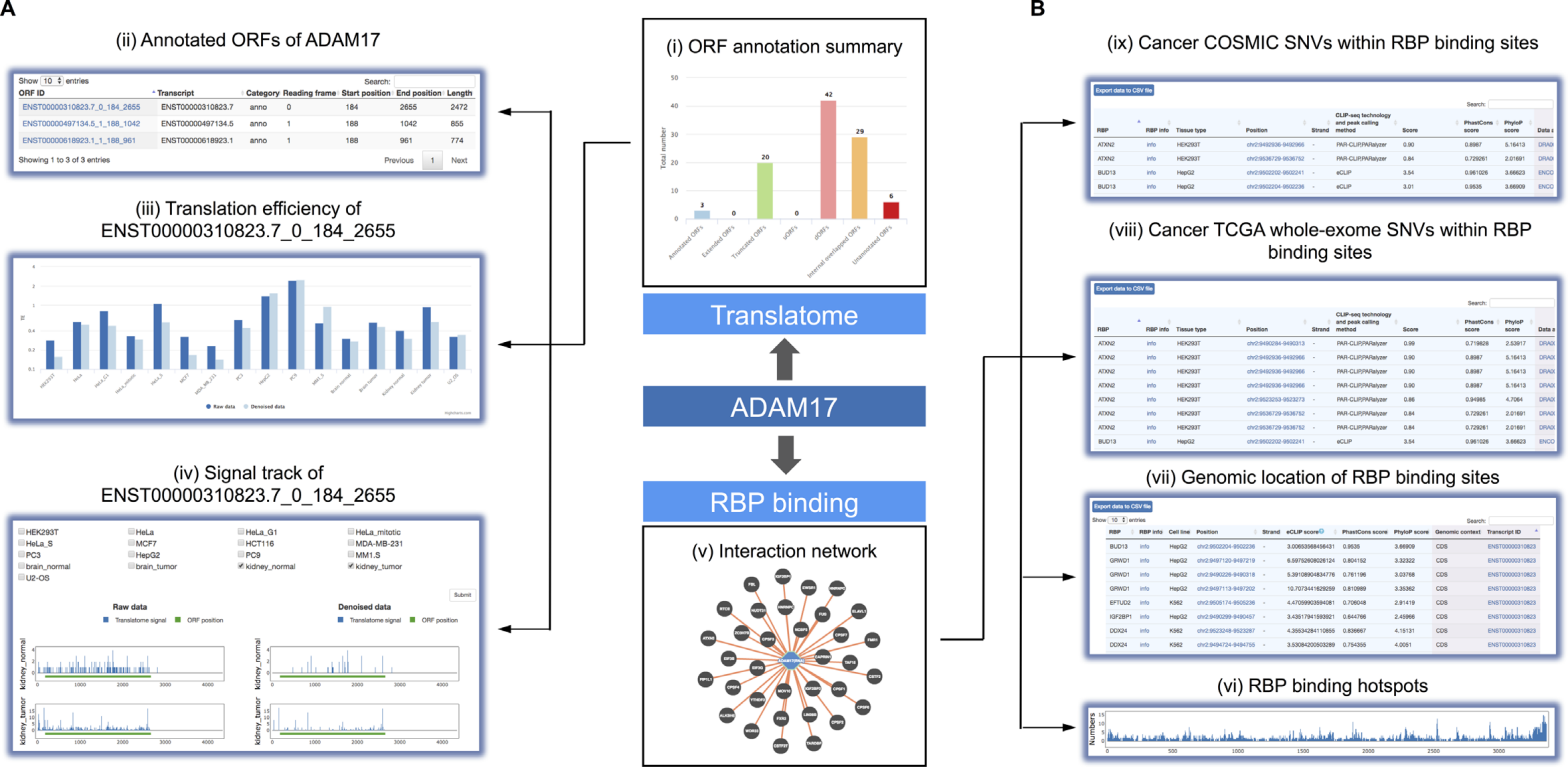
Integrative viewing of translation activity of a target gene (ADAM17) and its post-transcriptionally regulation events. (**A**) In the ‘Translatome’ module, all ORFs in ADAM17 are summarized based on their categories (i). Users can investigate each ORF by clicking on the name of the ORF (ii). For example, in ADAM17, estimation on the translation efficiency (iii) and the signal track (iv) reveals the potential of translation up-regulation in tumor samples compared to normal. (**B**) In the RBP module, search on ADAM17 provides the interactions network of ADAM17 gene and various RBPs (v). The number of RBPs binding along the transcript (vi) and genomic context of the binding sites (vii) can be visualized and searched. At last, the impact of SNVs in RBP-binding sites in both TCGA (viii) and COSMIC (ix) datasets further supports the association between ADAM17 and tumorigenesis.

ADAM17 encodes a membrane-bound protease and previous study demonstrate its role in tumorigenesis and invasiveness especially breast cancer (43). We observed overexpression of ADAM17 across most tumor samples compared with normal tissues using TCGA expression data (44). However, ADAM17 expression at protein level and the potential regulatory mechanism remains unexplored. We queried ‘ADAM17’ in the ‘Translatome’ module, POSTAR2 returned a histogram showing the numbers of categorized ORFs of ADAM17. Users can click on the ORF IDs for more details. Estimation on translation efficiency and signal track reveals the up-regulation at translation level in tumor samples compared to normal. For instance, both raw data and denoised data showed up-regulated translation efficiency in tumor tissue compared to paired normal tissue of brain and kidney (Figure 3A). To understand the potential mechanism that contribute to overexpression of ADAM17 at transcriptional and translational level, POSTAR2 shed light on RBP’s role in the regulatory mechanism. In the ‘RNA’ module, lots of RBP-binding sites identified by different CLIP-seq, the interaction network and RBP-binding hotspots represents numbers of RBP involved in the regulation of ADAM17 (Figure 3B). Among these RBPs, some RBPs such as EIF3B, EIF3G and EIF4A3 are the components of eukaryotic translation factor complex, which suggests that the interaction of these RBPs may participate in the translation regulatory of ADAM17. In addition, RBPs like FUS, TARDBP and ELAVL1 may contribute to the RNAs’ stability, which results in the aberrant expression level of RNAs or proteins. In addition, the output of the ‘Disease’ sub-module shows that lots of cancer mutations locate in the RBP-binding region on ADAM17, especially in kidney tumor and brain tumor.

## DISCUSSION AND FUTURE DIRECTIONS

POSTAR2 aims to decipher the post-transcriptional regulatory logics by integrating large-scale high-throughput sequencing datasets and other public resources. To our knowledge, POSTAR2 hosts the largest collection (∼40 million) of RBP-binding sites identified from CLIP-seq experiments, and enables the exploration for RNA–protein interactions with other post-transcriptional regulatory events and genomic variations. Moreover, Ribo-seq data were incorporated and analyzed to reveal the translational dynamics of RNAs. POSTAR2 enables integrated navigation of RBP-binding sites with multi-layer information of post-transcriptional regulation, phenotypes, diseases, as well as translational landscapes of RNAs.

In comparison with our previous version of POSTAR, POSTAR2 has the following novel features and improvements: (i) POSTAR2 integrates more CLIP-seq datasets from human and mouse. (ii) POSTAR2 includes CLIP-seq datasets from more species, including fly, worm, *Arabidopsis* and yeast. In total, we added and updated ∼500 CLIP-seq datasets in POSTAR2. (iii) POSTAR2 has a new module ‘Translatome’, which provides ∼36 million ORFs in the genomes from the six species. (iv) POSTAR2 annotates the RBP-binding sites with updated functional data resource. For example, we updated ∼1 million RNA modification sites and RNA editing sites curated from other databases and publications (45–47); updated and added ∼20 million SNPs from the genomes of the six species (48), as well as latest results of mutation-calling for TCGA samples (49). Finally, POSTAR2 provides an updated interactive interface to facilitate the investigation and exploration of RNA–protein interactions and translational landscape.

As advances in high-throughput sequencing technologies, CLIP-seq and Ribo-seq technologies will be applied to more cell and tissue types in more species, and more functional genomics datasets will be generated. We will continue to integrate new incoming data and improve the web interface for navigation and visualization. We will maintain and keep updating POSTAR2 to ensure it remains a valuable resource for the research community.

## DATA AVAILABILITY

POSTAR2 is freely available at http://lulab.life.tsinghua.edu.cn/postar. The datasets in POSTAR2 can be download and used in accordance with the GNU Public License and the license of their primary data sources.

## Supplementary Material

Supplementary Data

## References

[1] Hentze M.W., Castello A., Schwarzl T., Preiss T. A brave new world of RNA-binding proteins. Nat. Rev. Mol. Cell Biol. 2018; 19:327–341. 29339797 10.1038/nrm.2017.130

[2] Harvey R.F., Smith T.S., Mulroney T., Queiroz R.M.L., Pizzinga M., Dezi V., Villenueva E., Ramakrishna M., Lilley K.S., Willis A.E. Trans-acting translational regulatory RNA binding proteins. Wiley Interdiscip. Rev. RNA. 2018; 9:e1465. 29341429 10.1002/wrna.1465 PMC5947564

[3] Pereira B., Billaud M., Almeida R. RNA-Binding proteins in Cancer: Old players and new actors. Trends Cancer. 2017; 3:506–528. 28718405 10.1016/j.trecan.2017.05.003

[4] Lee F.C.Y., Ule J. Advances in CLIP technologies for studies of Protein-RNA interactions. Mol. Cell. 2018; 69:354–369. 29395060 10.1016/j.molcel.2018.01.005

[5] Konig J., Zarnack K., Luscombe N.M., Ule J. Protein-RNA interactions: new genomic technologies and perspectives. Nat. Rev. Genet. 2012; 13:77–83. 22251872 10.1038/nrg3141

[6] Ingolia N.T., Ghaemmaghami S., Newman J.R., Weissman J.S. Genome-wide analysis in vivo of translation with nucleotide resolution using ribosome profiling. Science. 2009; 324:218–223. 19213877 10.1126/science.1168978 PMC2746483

[7] Yang Y.C., Di C., Hu B., Zhou M., Liu Y., Song N., Li Y., Umetsu J., Lu Z.J. CLIPdb: a CLIP-seq database for protein-RNA interactions. BMC Genomics. 2015; 16:51. 25652745 10.1186/s12864-015-1273-2 PMC4326514

[8] Li J.H., Liu S., Zhou H., Qu L.H., Yang J.H. starBase v2.0: decoding miRNA-ceRNA, miRNA-ncRNA and protein-RNA interaction networks from large-scale CLIP-Seq data. Nucleic Acids Res. 2014; 42:D92–D97. 24297251 10.1093/nar/gkt1248 PMC3964941

[9] Calviello L., Ohler U. Beyond Read-Counts: Ribo-seq data analysis to understand the functions of the transcriptome. Trends Genet. 2017; 33:728–744. 28887026 10.1016/j.tig.2017.08.003

[10] Babitzke P., Baker C.S., Romeo T. Regulation of translation initiation by RNA binding proteins. Annu. Rev. Microbiol. 2009; 63:27–44. 19385727 10.1146/annurev.micro.091208.073514 PMC4682898

[11] Garcia-Maurino S.M., Rivero-Rodriguez F., Velazquez-Cruz A., Hernandez-Vellisca M., Diaz-Quintana A., De la Rosa M.A., Diaz-Moreno I. RNA Binding Protein Regulation and Cross-Talk in the Control of AU-rich mRNA Fate. Front. Mol. Biosci. 2017; 4:71. 29109951 10.3389/fmolb.2017.00071 PMC5660096

[12] Hu B., Yang Y.T., Huang Y., Zhu Y., Lu Z.J. POSTAR: a platform for exploring post-transcriptional regulation coordinated by RNA-binding proteins. Nucleic Acids Res. 2017; 45:D104–D114. 28053162 10.1093/nar/gkw888 PMC5210617

[13] Barrett T., Wilhite S.E., Ledoux P., Evangelista C., Kim I.F., Tomashevsky M., Marshall K.A., Phillippy K.H., Sherman P.M., Holko M. NCBI GEO: archive for functional genomics data sets–update. Nucleic Acids Res. 2013; 41:D991–D995. 23193258 10.1093/nar/gks1193 PMC3531084

[14] Davis C.A., Hitz B.C., Sloan C.A., Chan E.T., Davidson J.M., Gabdank I., Hilton J.A., Jain K., Baymuradov U.K., Narayanan A.K. The Encyclopedia of DNA elements (ENCODE): data portal update. Nucleic Acids Res. 2018; 46:D794–D801. 29126249 10.1093/nar/gkx1081 PMC5753278

[15] Van Nostrand E.L., Pratt G.A., Shishkin A.A., Gelboin-Burkhart C., Fang M.Y., Sundararaman B., Blue S.M., Nguyen T.B., Surka C., Elkins K. Robust transcriptome-wide discovery of RNA-binding protein binding sites with enhanced CLIP (eCLIP). Nat. Methods. 2016; 13:508–514. 27018577 10.1038/nmeth.3810 PMC4887338

[16] Langmead B., Trapnell C., Pop M., Salzberg S.L. Ultrafast and memory-efficient alignment of short DNA sequences to the human genome. Genome Biol. 2009; 10:R25. 19261174 10.1186/gb-2009-10-3-r25 PMC2690996

[17] Uren P.J., Bahrami-Samani E., Burns S.C., Qiao M., Karginov F.V., Hodges E., Hannon G.J., Sanford J.R., Penalva L.O., Smith A.D. Site identification in high-throughput RNA-protein interaction data. Bioinformatics. 2012; 28:3013–3020. 23024010 10.1093/bioinformatics/bts569 PMC3509493

[18] Corcoran D.L., Georgiev S., Mukherjee N., Gottwein E., Skalsky R.L., Keene J.D., Ohler U. PARalyzer: definition of RNA binding sites from PAR-CLIP short-read sequence data. Genome Biol. 2011; 12:R79. 21851591 10.1186/gb-2011-12-8-r79 PMC3302668

[19] Moore M.J., Zhang C., Gantman E.C., Mele A., Darnell J.C., Darnell R.B. Mapping Argonaute and conventional RNA-binding protein interactions with RNA at single-nucleotide resolution using HITS-CLIP and CIMS analysis. Nat. Protoc. 2014; 9:263–293. 24407355 10.1038/nprot.2014.012 PMC4156013

[20] Weyn-Vanhentenryck S.M., Mele A., Yan Q., Sun S., Farny N., Zhang Z., Xue C., Herre M., Silver P.A., Zhang M.Q. HITS-CLIP and integrative modeling define the Rbfox splicing-regulatory network linked to brain development and autism. Cell Rep. 2014; 6:1139–1152. 24613350 10.1016/j.celrep.2014.02.005 PMC3992522

[21] Finn R.D., Coggill P., Eberhardt R.Y., Eddy S.R., Mistry J., Mitchell A.L., Potter S.C., Punta M., Qureshi M., Sangrador-Vegas A. The Pfam protein families database: towards a more sustainable future. Nucleic Acids Res. 2016; 44:D279–D285. 26673716 10.1093/nar/gkv1344 PMC4702930

[22] Carbon S., Ireland A., Mungall C.J., Shu S., Marshall B., Lewis S., Ami G.O.H. the Web Presence Working Group AmiGO: online access to ontology and annotation data. Bioinformatics. 2009; 25:288–289. 19033274 10.1093/bioinformatics/btn615 PMC2639003

[23] Harrow J., Frankish A., Gonzalez J.M., Tapanari E., Diekhans M., Kokocinski F., Aken B.L., Barrell D., Zadissa A., Searle S GENCODE: the reference human genome annotation for The ENCODE Project. Genome Res. 2012; 22:1760–1774. 22955987 10.1101/gr.135350.111 PMC3431492

[24] dos Santos G., Schroeder A.J., Goodman J.L., Strelets V.B., Crosby M.A., Thurmond J., Emmert D.B., Gelbart W.M., FlyBase C. FlyBase: introduction of the Drosophila melanogaster Release 6 reference genome assembly and large-scale migration of genome annotations. Nucleic Acids Res. 2015; 43:D690–D697. 25398896 10.1093/nar/gku1099 PMC4383921

[25] Harris T.W., Antoshechkin I., Bieri T., Blasiar D., Chan J., Chen W.J., De La Cruz N., Davis P., Duesbury M., Fang R. WormBase: a comprehensive resource for nematode research. Nucleic Acids Res. 2010; 38:D463–D467. 19910365 10.1093/nar/gkp952 PMC2808986

[26] Rhee S.Y., Beavis W., Berardini T.Z., Chen G., Dixon D., Doyle A., Garcia-Hernandez M., Huala E., Lander G., Montoya M. The Arabidopsis Information Resource (TAIR): a model organism database providing a centralized, curated gateway to Arabidopsis biology, research materials and community. Nucleic Acids Res. 2003; 31:224–228. 12519987 10.1093/nar/gkg076 PMC165523

[27] Cherry J.M., Hong E.L., Amundsen C., Balakrishnan R., Binkley G., Chan E.T., Christie K.R., Costanzo M.C., Dwight S.S., Engel S.R. Saccharomyces Genome Database: the genomics resource of budding yeast. Nucleic Acids Res. 2012; 40:D700–D705. 22110037 10.1093/nar/gkr1029 PMC3245034

[28] Zhao Y., Li H., Fang S., Kang Y., Wu W., Hao Y., Li Z., Bu D., Sun N., Zhang M.Q. NONCODE 2016: an informative and valuable data source of long non-coding RNAs. Nucleic Acids Res. 2016; 44:D203–D208. 26586799 10.1093/nar/gkv1252 PMC4702886

[29] Trapnell C., Pachter L., Salzberg S.L. TopHat: discovering splice junctions with RNA-Seq. Bioinformatics. 2009; 25:1105–1111. 19289445 10.1093/bioinformatics/btp120 PMC2672628

[30] Trapnell C., Williams B.A., Pertea G., Mortazavi A., Kwan G., van Baren M.J., Salzberg S.L., Wold B.J., Pachter L. Transcript assembly and quantification by RNA-Seq reveals unannotated transcripts and isoform switching during cell differentiation. Nat. Biotechnol. 2010; 28:511–515. 20436464 10.1038/nbt.1621 PMC3146043

[31] Papatheodorou I., Fonseca N.A., Keays M., Tang Y.A., Barrera E., Bazant W., Burke M., Fullgrabe A., Fuentes A.M., George N. Expression Atlas: gene and protein expression across multiple studies and organisms. Nucleic Acids Res. 2018; 46:D246–D251. 29165655 10.1093/nar/gkx1158 PMC5753389

[32] Yang Y., Umetsu J., Lu Z.J. Global signatures of protein binding on structured RNAs in Saccharomyces cerevisiae. Sci. China Life sci. 2014; 57:22–35. 24369346 10.1007/s11427-013-4583-0

[33] Bailey T.L., Elkan C. Fitting a mixture model by expectation maximization to discover motifs in biopolymers. Proc. Int. Conf. Intell. Syst. Mol. Biol. 1994; 2:28–36. 7584402

[34] Heinz S., Benner C., Spann N., Bertolino E., Lin Y.C., Laslo P., Cheng J.X., Murre C., Singh H., Glass C.K. Simple combinations of lineage-determining transcription factors prime cis-regulatory elements required for macrophage and B cell identities. Mol. Cell. 2010; 38:576–589. 20513432 10.1016/j.molcel.2010.05.004 PMC2898526

[35] Grant C.E., Bailey T.L., Noble W.S. FIMO: scanning for occurrences of a given motif. Bioinformatics. 2011; 27:1017–1018. 21330290 10.1093/bioinformatics/btr064 PMC3065696

[36] Crooks G.E., Hon G., Chandonia J.M., Brenner S.E. WebLogo: a sequence logo generator. Genome Res. 2004; 14:1188–1190. 15173120 10.1101/gr.849004 PMC419797

[37] Kazan H., Ray D., Chan E.T., Hughes T.R., Morris Q. RNAcontext: a new method for learning the sequence and structure binding preferences of RNA-binding proteins. PLoS Comput. Biol. 2010; 6:e1000832. 20617199 10.1371/journal.pcbi.1000832 PMC2895634

[38] Rabani M., Kertesz M., Segal E. Computational prediction of RNA structural motifs involved in posttranscriptional regulatory processes. Proc. Natl. Acad. Sci. U.S.A. 2008; 105:14885–14890. 18815376 10.1073/pnas.0803169105 PMC2567462

[39] Xu Z., Hu L., Shi B., Geng S., Xu L., Wang D., Lu Z.J. Ribosome elongating footprints denoised by wavelet transform comprehensively characterize dynamic cellular translation events. Nucleic Acids Res. 2018; doi:10.1093/nar/gky533. 10.1093/nar/gky533 PMC6182183 29945224

[40] Calviello L., Mukherjee N., Wyler E., Zauber H., Hirsekorn A., Selbach M., Landthaler M., Obermayer B., Ohler U. Detecting actively translated open reading frames in ribosome profiling data. Nat. Methods. 2016; 13:165–173. 26657557 10.1038/nmeth.3688

[41] Bazzini A.A., Johnstone T.G., Christiano R., Mackowiak S.D., Obermayer B., Fleming E.S., Vejnar C.E., Lee M.T., Rajewsky N., Walther T.C. Identification of small ORFs in vertebrates using ribosome footprinting and evolutionary conservation. EMBO J. 2014; 33:981–993. 24705786 10.1002/embj.201488411 PMC4193932

[42] Ji Z., Song R., Regev A., Struhl K. Many lncRNAs, 5′UTRs, and pseudogenes are translated and some are likely to express functional proteins. elife. 2015; 4:e08890. 26687005 10.7554/eLife.08890 PMC4739776

[43] McGowan P.M., Ryan B.M., Hill A.D., McDermott E., O’Higgins N., Duffy M.J. ADAM-17 expression in breast cancer correlates with variables of tumor progression. Clin. Cancer Res. 2007; 13:2335–2343. 17438092 10.1158/1078-0432.CCR-06-2092

[44] Tang Z., Li C., Kang B., Gao G., Li C., Zhang Z. GEPIA: a web server for cancer and normal gene expression profiling and interactive analyses. Nucleic Acids Res. 2017; 45:W98–W102. 28407145 10.1093/nar/gkx247 PMC5570223

[45] Dominissini D., Nachtergaele S., Moshitch-Moshkovitz S., Peer E., Kol N., Ben-Haim M.S., Dai Q., Di Segni A., Salmon-Divon M., Clark W.C. The dynamic N(1)-methyladenosine methylome in eukaryotic messenger RNA. Nature. 2016; 530:441–446. 26863196 10.1038/nature16998 PMC4842015

[46] Xuan J.J., Sun W.J., Lin P.H., Zhou K.R., Liu S., Zheng L.L., Qu L.H., Yang J.H. RMBase v2.0: deciphering the map of RNA modifications from epitranscriptome sequencing data. Nucleic Acids Res. 2018; 46:D327–D334. 29040692 10.1093/nar/gkx934 PMC5753293

[47] Zhao H.Q., Zhang P., Gao H., He X., Dou Y., Huang A.Y., Liu X.M., Ye A.Y., Dong M.Q., Wei L. Profiling the RNA editomes of wild-type C. elegans and ADAR mutants. Genome Res. 2015; 25:66–75. 25373143 10.1101/gr.176107.114 PMC4317174

[48] Sherry S.T., Ward M.H., Kholodov M., Baker J., Phan L., Smigielski E.M., Sirotkin K. dbSNP: the NCBI database of genetic variation. Nucleic Acids Res. 2001; 29:308–311. 11125122 10.1093/nar/29.1.308 PMC29783

[49] Ellrott K., Bailey M.H., Saksena G., Covington K.R., Kandoth C., Stewart C., Hess J., Ma S., Chiotti K.E., McLellan M. Scalable open science approach for mutation calling of tumor exomes using multiple genomic pipelines. Cell Syst. 2018; 6:271–281. 29596782 10.1016/j.cels.2018.03.002 PMC6075717

